# KIZ/GM114 Balances the NF-ĸB Signaling by Antagonizing the Association of TRAF2/6 With Their Upstream Adaptors

**DOI:** 10.3389/fcell.2022.877039

**Published:** 2022-03-31

**Authors:** Jiawei Sun, Qili Yang, Enping Liu, Dahua Chen, Qinmiao Sun

**Affiliations:** ^1^ State Key Laboratory of Membrane Biology, Institute of Zoology, Chinese Academy of Sciences, Beijing, China; ^2^ Institute of Stem Cells and Regeneration, Chinese Academy of Sciences, Beijing, China; ^3^ School of Life Sciences, University of Chinese Academy of Sciences, Beijing, China; ^4^ Institute of Biomedical Research, Yunnan University, Kunming, China

**Keywords:** NF-κB signaling, TRAF6/2, KIZ, homeostasis of inflammatory response, inflammatory bowel disease

## Abstract

NF-κB signaling is a pivotal regulator of the inflammatory response and it must be tightly controlled to avoid an excessive inflammatory response that may lead to human chronic inflammatory and autoimmune diseases. Thus, how NF-κB signaling is precisely controlled is a long-standing question in the field. TRAF family proteins function as key adaptors to mediate NF-κB signaling induced by various receptors. Here, we characterize KIZ/GM114 as a negative regulator balancing the NF-κB signaling. Mechanistically, KIZ/GM114 binds TRAF6/2 by targeting the TRAF domains to antagonize the TRAF6-IRAK1 association or the TRAF2-TRADD association, consequently reducing the IL-1β/LPS/TNFα-induced NF-κB activation. Importantly, upon dextran sulfate sodium treatment, *Gm114* deficiency induces a stronger inflammatory response, more severe acute colitis and lower survival rate in mice compared with control mice. Collectively, our study not only identifies KIZ/GM114 as a negative regulator to balance the NF-κB signaling, but it also implies a new strategy for limiting excessive inflammatory response.

## Introduction

Nuclear factor-κB (NF-κB) signaling plays a critical role in regulating the inflammatory response by modulating the expression of inflammatory proteins and proinflammatory cytokines (Popa et al., 2007; Lawrence 2009). Various inflammatory stimuli, which include lipopolysaccharide (LPS), IL-1β, and TNFα, trigger NF-κB activation (T. Liu et al., 2017). IL-1β and LPS bind to IL-1 receptor (IL-1R) and Toll-like receptor (TLR) 4, respectively, and they share many common components to activate NF-κB signaling pathway. In these two pathways, after binding to IL-1β/LPS, the receptors recruit adaptor protein MyD88 through their respective TIR domain and then MyD88 recruits IRAKs that include IRAK4 and IRAK1 (Takeda and Akira 2004; Kawai and Akira 2010; Moresco et al., 2011). IRAK1 is phosphorylated by IRAK4 and then associates with TRAF6, an E3 ubiquitin ligase that functions together with the Ubc13/Uev1A complex to catalyze the synthesis of a polyubiquitin chain linked through Lys-63 (K63) of ubiquitin (Cao et al., 1996; Xia et al., 2009; Deng et al., 2000; Z. J.; Chen 2012). The K63-linked polyubiquitin chains bind to TAB2 and NEMO, and then activate the TAK1 complex (TAK1, TAB1, and TAB2/3) and IKK complex (IKKα, IKKβ, and NEMO), respectively (Wang et al., 2001; Kanayama et al., 2004; Wu et al., 2006; Ajibade, Wang, and Wang 2013). Activated IKKβ further phosphorylates IκBα, which results in its degradation by the proteasome and the release of NF-κB from IκBα. Then, NF-κB enters the nucleus to regulate the expression of downstream genes such as proinflammatory cytokines IL-6, IL-1β, TNFα, and IL-8 (Hayden and Ghosh 2008; Oeckinghaus et al., 2011). In TNFα-mediated NF-κB signaling, upon TNFα binding, tumor necrosis factor receptor (TNFR) 1 recruit the adaptor protein TRADD through their respective death domains (Hsu et al., 1995). TRADD further recruits TRAF2, cIAP1, cIAP2, and RIP1 to form a complex, where RIP1 undergoes K63-linked ubiquitination, which leads to activation of the TAK1 complex, and subsequently inducing NF-κB signaling (Hayden and Ghosh 2014; Ea et al., 2006; Li et al., 2006; Hsu et al., 1996b; Hsu et al., 1996a; Y. C; Park et al., 2000). Considering that NF-κB activation is critical for inflammatory responses, it must be tightly regulated to avoid excessive inflammation. Uncontrolled activation of the NF-κB signaling promotes chronic inflammation and autoimmune diseases such as inflammatory bowel disease, arthritis, and obesity (Cook et al., 2004; Marshak-Rothstein 2006; Simmonds and Foxwell 2008). Therefore, it is very important to understand the mechanisms that maintain NF-κB signaling to ensure immune homeostasis.

Tumor necrosis factor receptor–associated factor (TRAF) proteins function as key signaling molecules to mediate a variety of receptor-signaling pathways that involving in immune response, development and diseases (Inoue et al., 2000; Xie 2013a). Typically, TRAF proteins have two major functions: the E3 ligase and scaffolding functions (H. H. Park 2018; Walsh et al., 2015). The E3 ligase function of TRAF protein relies on the N-terminal RING Finger domain, while the scaffolding function is achieved through the C-terminal TRAF domain. The TRAF domain in TRAF proteins has also been reported to mediate interactions with upstream regulators and downstream effectors (Inoue et al., 2000; Wajant et al., 2001; Ha et al., 2009). We recently reported that *Drosophila* Bam forms a complex with Otu by targeting dTRAF6 for the cleavage of its K63-linked ubiquitin chains. Thus, Otu/Bam regulates immune deficiency (IMD) signaling to maintain gut immune homeostasis, thereby controlling the fly lifespan (Ji et al., 2019). Of note, both Toll and IMD signaling pathways in *Drosophila* have similar components to TLR and TNFR signaling pathways in mammals, respectively, both of which mediate NF-κB activation (Hetru and Hoffmann 2009). Mouse GM114 has been reported to be a putative homolog of Bam with relatively low sequence similarities. However, in contrast to the function of Bam, GM114 is dispensable for germline development (McKearin and Spradling 1990; Tang et al., 2008). In this study, we evaluated the roles of GM114 and KIZ (GM114 human homolog) in the innate immune response and found that GM114/KIZ played a negative role in regulating NF-κB signaling induced by IL-1β/LPS and TNFα. Mechanistically, GM114/KIZ suppressed IL-1β/LPS-triggered NF-κB signaling by interacting with TRAF6, which antagonized the association of TRAF6 with IRAK1, while GM114/KIZ attenuated TNFα-induced NF-κB signaling by interacting with TRAF2, which subsequently decreased the association of TRAF2 with TRADD. Moreover, *Gm114*-deficient mice were more susceptible to acute colitis induced by dextran sulfate sodium (DSS) compared with wild-type mice because of higher inflammatory responses.

## Results

### Overexpression of KIZ Reduces NF-ĸB Activation Triggered by IL-1β/LPS and TNFα

**FIGURE 1:**
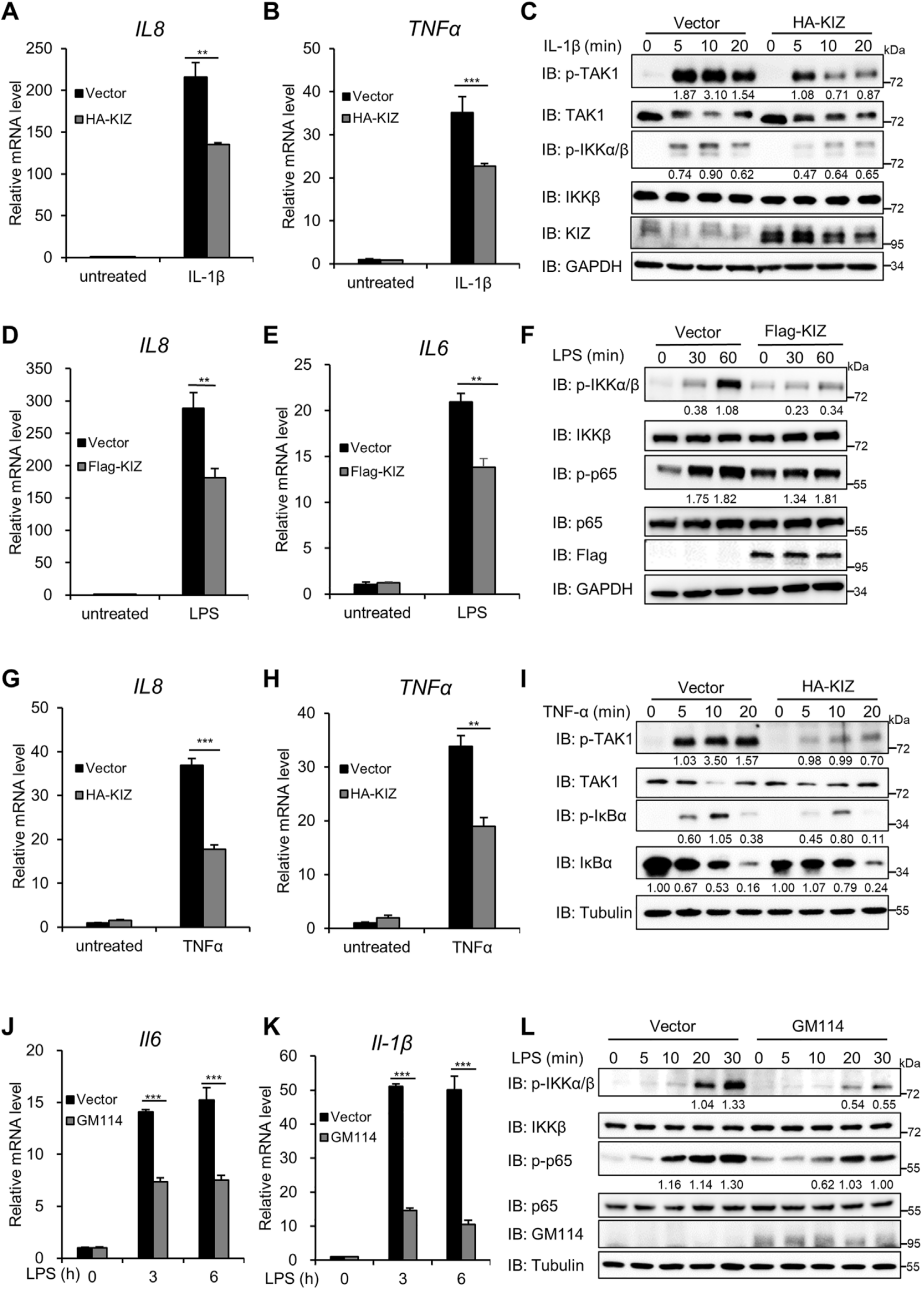
Overexpression of KIZ/GM114 decreases IL-1β/LPS/TNFα- induced NF-κB activation. **(A–C)** HEK293 C6 cells were transfected with an empty vector or HA-tagged KIZ. After 24 h of transfection, the cells were stimulated with human IL-1β (5 ng/ml) for 3 h and then analyzed by qPCR to measure the mRNA levels of *IL8* **(A)** and *TNFα* **(B)** or stimulated for the indicated times and analyzed by immunoblotting with the indicated antibodies **(C)**. **(D–F)** THP1 cells were infected with a lentivirus that expressed an empty vector or KIZ for 48 h. The cells were then stimulated with LPS (1 μg/ml) for 3 h, followed by qPCR to measure the mRNA levels of *IL8* **(D)** and *IL6* **(E)** or stimulated for the indicated times, followed by immunoblotting **(F)**. **(G–I)** Similar to **(A–C)**, except the cells were stimulated with human TNFα (20 ng/ml). **(J–L)** RAW264.7 cells were infected with a lentivirus that expressed an empty vector or GM114 for 48 h, stimulated with LPS (100 ng/ml) for the indicated times, and then analyzed by qPCR to measure the mRNA levels of *Il6* **(J)** and *Il-1β* **(K)** or subjected to immunoblotting **(L)**. Data shown in **(A,B,D,E,G,H,J,K)** are from one representative experiment of three independent experiments (mean ± SD, *n* = 3). **p < 0.01; ***p < 0.001; two-tailed Student’s t-test.

Proper regulation of NF-κB signal transduction is important for cellular functions. Therefore, we searched for factors that maintain homeostasis of the NF-κB pathway. We recently showed that *Drosophila* Bam played a role in regulating IMD signaling. Considering that GM114 is a putative mouse homolog of *Drosophila* Bam, we investigated whether GM114 and its human homolog KIZ also contributed to regulating the NF-κB signaling pathway in mammals. First, we overexpressed KIZ or the empty vector in C6 cells that ectopically express IL-1R and then stimulated the cells with IL-1β, followed by quantitative PCR (qPCR) and immunoblotting. qPCR showed that KIZ overexpression significantly reduced the transcriptional levels of IL-1β-induced NF-κB downstream genes that included *IL8* and *TNFα* (Figures 1A,B). Consistently, immunoblotting showed that the levels of phosphorylated TAK1 and IKKα/β induced by IL-1β were dramatically decreased in KIZ-overexpressing cells (Figure 1C). Because both LPS and IL-1β trigger NF-κB activation and share very similar intracellular signaling cascades, we next examined whether KIZ mediated LPS-induced NF-κB signaling in human macrophage THP1 cells. As shown in Figures 1D,E, the mRNA levels of *IL8* and *IL6* induced by LPS were significantly lower in KIZ-overexpressing cells than in the control cells. Immunoblotting also demonstrated that the levels of phosphorylated IKKα/β and p65 induced by LPS were remarkedly reduced in KIZ-overexpressing cells (Figure 1F). To determinate whether KIZ specifically modulated NF-κB signaling induced by LPS/IL-1β, KIZ was overexpressed in C6 cells, then the cells were stimulated by TNFα. Similarly, qPCR showed that KIZ overexpression attenuated the mRNA levels of *IL8* and *TNFα* induced by TNFα (Figures 1G,H) and immunoblotting consistently demonstrated that KIZ overexpression decreased the levels of phosphorylated TAK1 and IκBα induced by TNFα (Figure 1I). These data suggest that KIZ is involved in regulating NF-κB signaling induced by IL-1β/LPS and TNFα. Next, we examined whether GM114 plays a conserved role in NF-κB signaling in mouse cells. As shown in Figures 1J,K, ectopic expression of GM114 markedly decreased the mRNA levels of *Il6* and *Il1β* induced by LPS in mouse macrophage RAW264.7 cells. Consistently, GM114 overexpression decreased the levels of phosphorylated IKKα/β and p65 induced by LPS (Figure 1L). Collectively, these findings demonstrate that KIZ/GM114 plays a conserved negative role in regulating NF-κB activation induced by IL-1β/LPS and TNFα.

### Knockdown of KIZ Augments NF-κB Signaling Induced by IL-1β/LPS and TNFα

**FIGURE 2:**
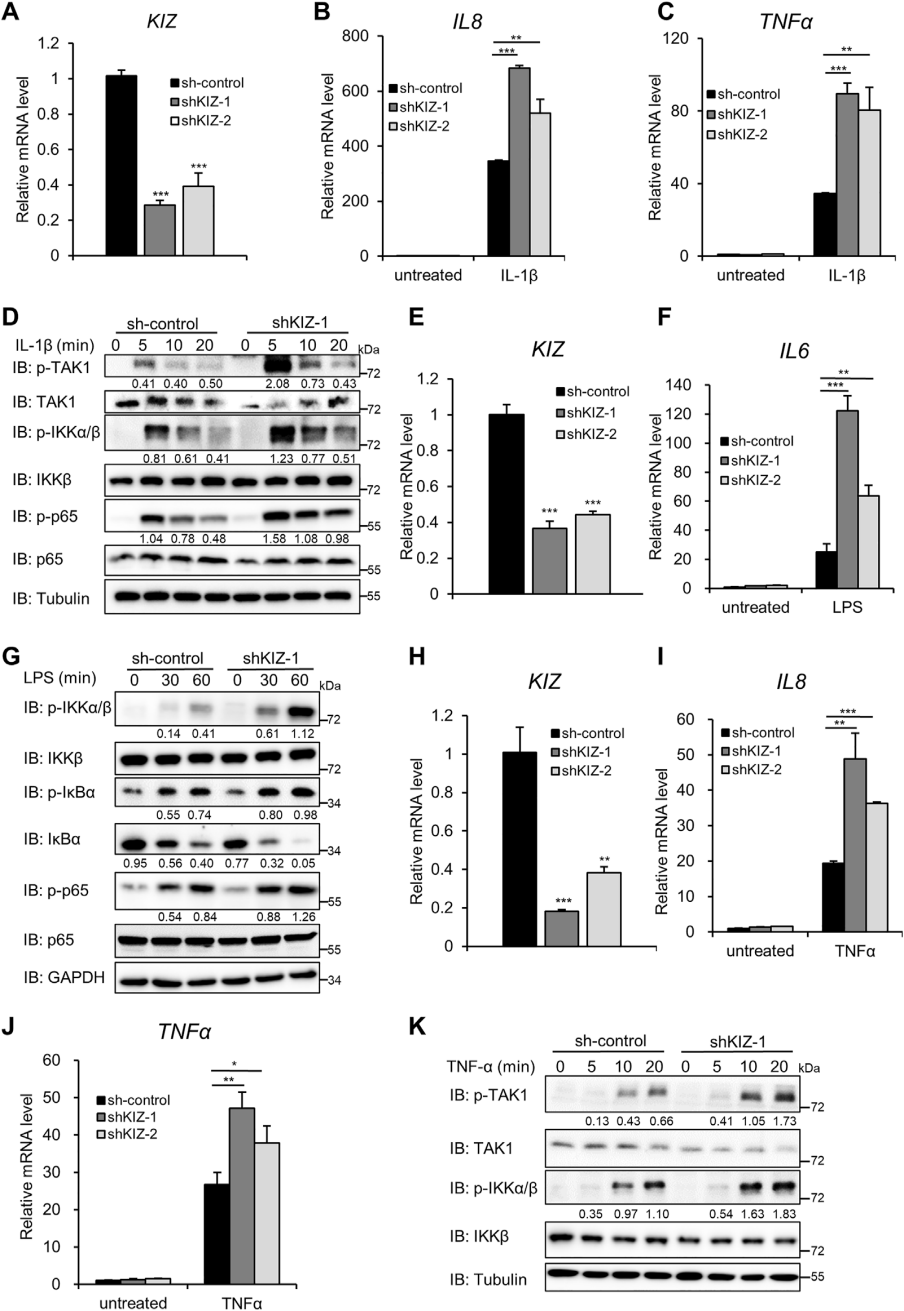
Knockdown of KIZ enhances IL-1β/LPS/TNFα-induced NF-κB activation. **(A–D)** HeLa cells were infected with an shRNA lentivirus that targeted different coding regions of *KIZ* (shKIZ-1 and shKIZ-2) or the empty vector for 48 h. The cells were then untreated or treated with IL-1β (20 ng/ml) for 3 h and then analyzed by qPCR to measure the mRNA levels of *KIZ* **(A)**, *IL8* **(B)**, and *TNFα* **(C)** or treated for the indicated times, followed by immunoblotting **(D)**. **(E–G)** THP1 cells were infected with a lentivirus that targeted different coding regions of *KIZ* (shKIZ-1 and shKIZ-2) or the empty vector. After 48 h of infection, the cells were untreated or treated with LPS (1 μg/ml) for 3 h and then analyzed by qPCR to measure the mRNA levels of *KIZ* **(E)** and *IL6* **(F)** or treated for the indicated times, followed by immunoblotting **(G)**. **(H–K)** Similar to **(A–D)**, except the cells were treated with TNFα (20 ng/ml). Data shown in **(A–C,E,F,H–J)** are from one representative experiment of three independent experiments (mean ± SD, *n* = 3). *p < 0.05; **p < 0.01; ***p < 0.001; two-tailed Student’s t-test.

To further investigate the physiological role of KIZ in NF-κB signaling, we next employed lentivirus-delivered shRNAs that targeted two non-overlapping cDNA regions of human *KIZ* to knockdown *KIZ*. qPCR showed that both shKIZ-1 and shKIZ-2 efficiently reduced the amount of endogenous *KIZ* in HeLa cells (Figure 2A) and knockdown of *KIZ* significantly increased the mRNA levels *IL8* and *TNFα* induced by IL-1β (Figures 2B,C). Consistently, the levels of phosphorylated TAK1, IKKα/β, and p65 were substantially increased in *KIZ* knockdown cells after IL-1β treatment (Figure 2D). To confirm the function of KIZ, we carried out similar knockdown experiments in U2OS cells and obtained consistent results (Supplementary Figures S1A–D). Next, we investigated whether alteration of KIZ expression affected LPS-induced NF-κB signaling. As shown in Figures 2E–G, knockdown of KIZ had similar enhancing effects on the activation of NF-κB after LPS treatment in THP1 cells, while no difference was observed on the mRNA level of IRF3 downstream gene *IFIT1* (Supplementary Figure S1E), which indicated the specific role of KIZ in regulating NF-κB signaling. We also observed that, similar to IL-1β stimulation, knockdown of KIZ significantly augmented TNFα-induced mRNA levels of *IL8* and *TNFα* (Figures 2H–J). Immunoblotting also demonstrated that the levels of phosphorylated TAK1 and IKKα/β induced by TNFα were remarkedly enhanced in KIZ knockdown HeLa cells compared with the control cells (Figure 2K). Taken together, these results further support the idea that KIZ functions as a negative modulator of NF-κB activation induced by LPS/IL-1β and TNFα.

### KIZ/Gm114 Deficiency Enhances NF-ĸB Activation Induced by LPS/IL-1β and TNFα

**FIGURE 3:**
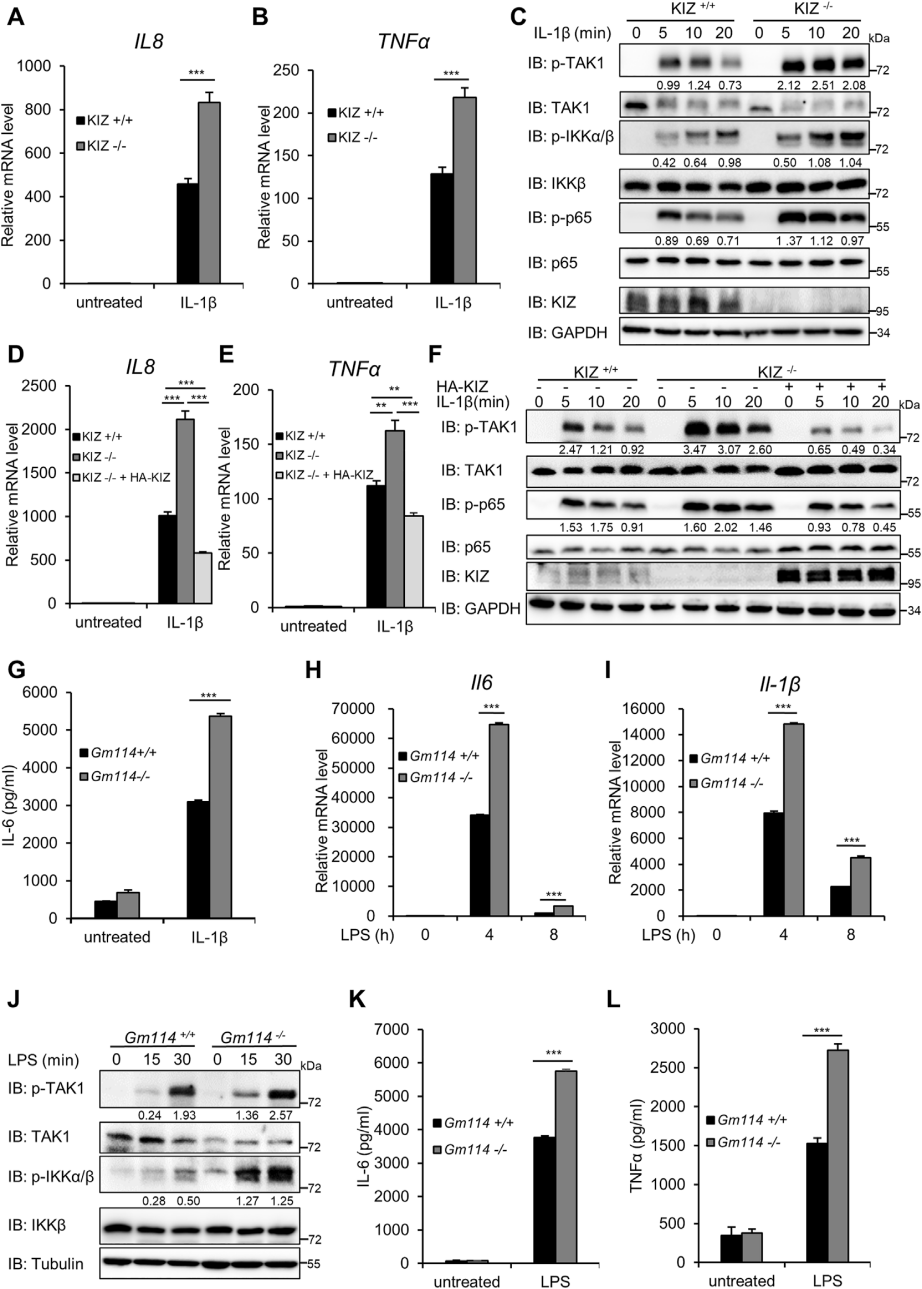
KIZ/Gm114 deficiency promotes IL-1β/LPS-induced NF-κB activation. **(A–C)** *KIZ* ^*+/+*^ and *KIZ* ^*−/−*^ HEK293 C6 cells were untreated or treated with IL-1β (5 ng/ml) for 3 h, followed by qPCR to measure the mRNA levels of *IL8* **(A)** and *TNFα* **(B)** or stimulated for the indicated times for immunoblotting **(C)**. **(D–F)** WT and KIZ knockout HEK293 C6 cells were transfected with the KIZ expression plasmid or empty vector as indicated. After 24 h, the cells were untreated or treated with IL-1β (5 ng/ml) for 3 h, followed by qPCR to measure the mRNA levels of *IL8* **(D)** and *TNFα* **(E)** or treated for the indicated times, followed by immunoblotting **(F)**. **(G)** *Gm114* ^*+/+*^ and *Gm114* ^*−/−*^ MEFs were treated with IL-1β (2 ng/ml) for 24 h and then analyzed by ELISA to measure IL-6 in the culture supernatant. **(H–J)** BMDMs were isolated from 8-week-old *Gm114* ^*+/+*^ and *Gm114* ^*−/−*^ mice, and untreated or treated with LPS (100 ng/ml) for the indicated times and then analyzed by qPCR to measure the mRNA levels of *Il6* **(H)** and *Il-1β* **(I)** or subjected to immunoblotting **(J)**. **(K,L)** *Gm114* ^*+/+*^ and *Gm114* ^*−/−*^ BMDMs were treated with LPS (100 ng/ml) for 24 h and then culture supernatants were collected to measure IL-6 **(K)** and TNFα **(L)** by ELISAs. Data shown in **(A,B,D,E,G–I,K,L)** are from one representative experiment of three independent experiments (mean ± SD, *n* = 3). **p < 0.01; ***p < 0.001; two-tailed Student’s t-test.

To confirm that endogenous KIZ/GM114 is required to regulate NF-ĸB signaling under physiological conditions, we generated *KIZ* knockout HEK293 C6 cells using the CRISPR-Cas9 approach. *KIZ* deficiency was confirmed by genomic sequencing and immunoblotting with an anti-KIZ antibody produced in-house (Supplementary Figure S2A). NF-κB activation in *KIZ* ^*−/−*^ and *KIZ* ^*+/+*^ cells was measured after IL-1β stimulation. The results in Figures 3A,B showed that *KIZ* deficiency significantly increased the mRNA levels of *IL8* and *TNFα* triggered by IL-1β compared with the control cells. Consistently, IL-1β-induced levels of phosphorylated TAK1, IKKα/β, and p65 were also elevated in *KIZ* ^−/*−*^ cells (Figure 3C). To confirm the specific role of KIZ in NF-κB signaling, we performed rescue experiments and found that restored expression of KIZ in *KIZ* ^−/*−*^ cells reversed the increased mRNA levels of *IL8* and *TNFα* induced by IL-1β (Figures 3D,E), as well as the levels of phosphorylated TAK1 and p65 (Figure 3F). Similarly, *KIZ* knockout cells also displayed enhanced TNFα-induced NF-κB activation (Supplementary Figures S2B–D). These findings further support the idea that KIZ is required to maintain the balance of NF-κB signaling.

To further validate the physiological roles of GM114 in the regulation of NF-κB signaling, we generated *Gm114* knockout mice on the C57BL6 background with deletion of exons 2 and 3 in mouse *Gm114* gene alleles (Supplementary Figure S3A). *Gm114* deletion was verified by the anti-GM114 antibody (Supplementary Figure S3B). GM114 homozygous null mice were born at the Mendelian ratio, viable, and fertile, and exhibited no detectable developmental defects, which were consistent with a previous study (Tang et al., 2008). To determine the effect of *Gm114* deficiency on NF-κB signaling, we isolated mouse embryonic fibroblasts (MEFs) from 13.5-day-old embryos of *Gm114* ^+/+^ and *Gm114* ^−/−^ mice by breeding heterozygous mutants and then stimulated the cells with IL-1β. As shown in Supplementary Figures S3C, D, the mRNA levels of *Il6* and *Il1β* were significantly enhanced in *Gm114* ^*−/−*^ MEFs compared with the WT controls in response to IL-1β stimulation. Consistently, immunoblotting showed that *Gm114* depletion increased the levels of phosphorylated IKKα/β and p65 induced by IL-1β (Supplementary Figure S3E). To confirm these results, we performed enzyme-linked immunosorbent assays (ELISAs) and found that secreted IL-6 induced by IL-1β was significantly higher in *Gm114* ^*−/−*^ MEFs than in the control cells (Figure 3G). Next, we stimulated bone marrow-derived macrophages (BMDMs) of wild-type and *Gm114* knockout mice with LPS and found that *Gm114* deficiency also significantly enhanced mRNA levels of *Il6* and *Il1β* (Figures 3H,I), and abundances of phosphorylated TAK1 and IKKα/β upon LPS treatment compared with the controls (Figure 3J). The results of ELISAs also showed that *Gm114* deficiency augmented the production of IL-6 and TNFα induced by LPS in BMDMs (Figures 3K,L). Additionally, *Gm114* knockout in MEFs also increased activation of NF-κB induced by TNFα (Supplementary Figures S3F–I). Taken together, these results further support that GM114/KIZ acts as a conserved negative regulator of NF-κB signaling.

### KIZ Targets TRAF6 to Regulate NF-ĸB Activation Induced by IL-1β

**FIGURE 4:**
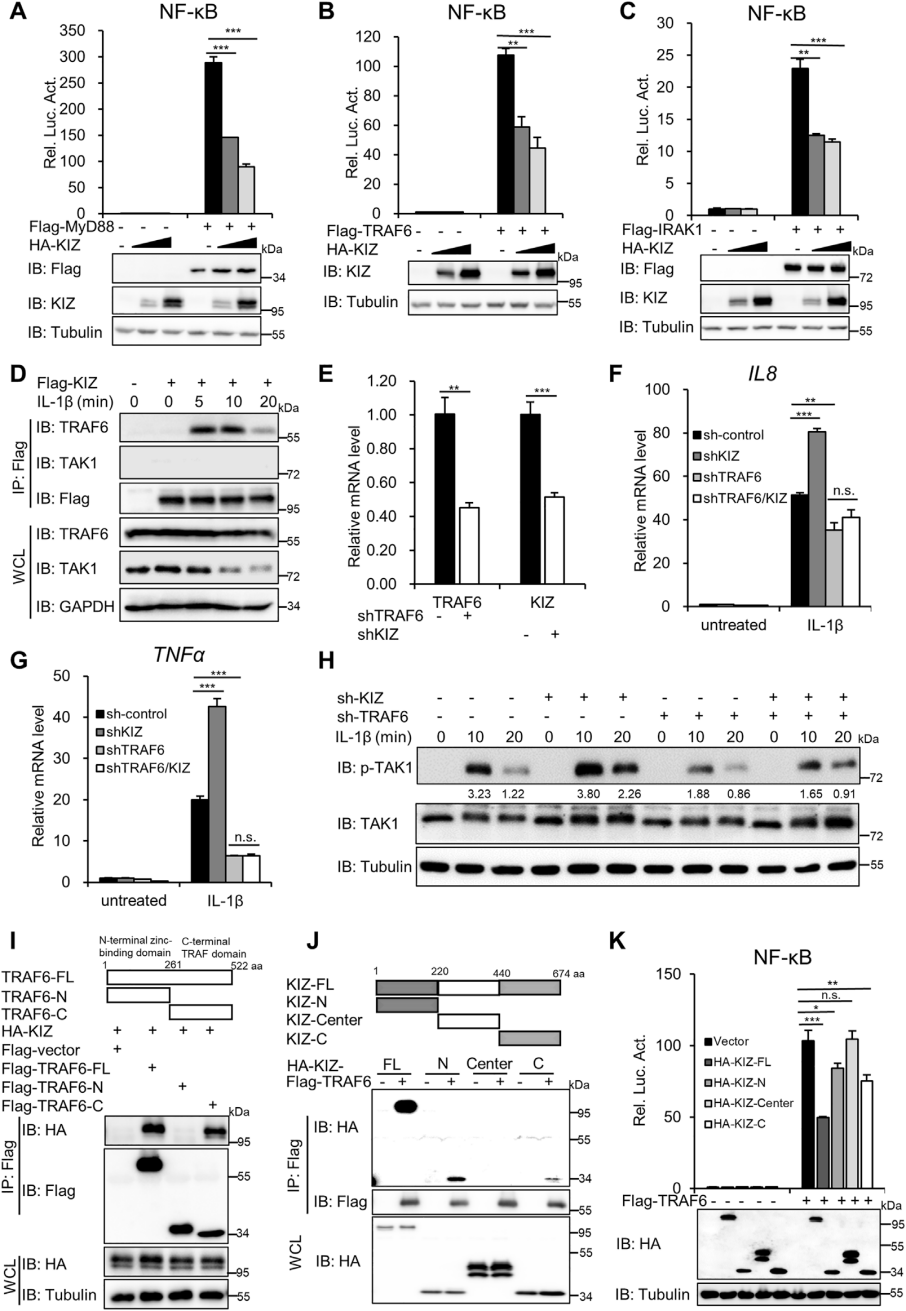
KIZ regulates IL-1β-induced NF-ĸB activation by targeting TRAF6. **(A–C)**. HEK293T cells were transfected with increasing doses of KIZ plasmids or an empty vector together with an NF-κB-Luc reporter and plasmids that expressed Flag-MyD88 **(A)**, Flag-TRAF6 **(B)**, or Flag-IRAK1 **(C)**. Twenty-four hours after transfection, the cells were lysed for luciferase reporter assays (upper panel) and immunoblotting (lower panels). **(D)** HEK293 C6 cells were transfected with a Flag-tagged KIZ expression plasmid. After 24 h, the cells were untreated or treated with IL-1β (5 ng/ml) for the indicated times, lysed for IP with anti-Flag beads, and then analyzed by immunoblotting. **(E–H)** HeLa cells were infected with a shRNA lentivirus that targeted *KIZ*, *TRAF6*, both, or the empty vector for 48 h. The cells were untreated or treated with IL-1β (20 ng/ml) for 3 h and then analyzed by qPCR to measure the mRNA levels of *KIZ*, *TRAF6* **(E)**, *IL8* **(F)**, and *TNFα* **(G)** or treated for the indicated times and then analyzed by immunoblotting **(H)**. **(I)** Schematic diagram of TRAF6 domains (upper panel). HEK293T cells were co-transfected with KIZ and TRAF6 or its truncated variants. After 24 h, the cells were lysed for IP with anti-Flag beads and analyzed by immunoblotting (lower panels). **(J)** Schematic diagram of KIZ and its truncated variants (upper panel). HEK293T cells were co-transfected with TRAF6 and KIZ or its truncated variants. After 24 h, the cells were lysed for IP with anti-Flag beads and analyzed by immunoblotting (lower panels). **(K)** HEK293T cells were transfected with Flag-TRAF6 or an empty vector together with a NF-κB-Luc reporter and plasmids that expressed HA-tagged KIZ or its truncated variants as indicated. After 24 h of transfection, the cells were lysed for luciferase reporter assays (upper panel) and immunoblotting (lower panels). Data shown in **(A–C,E–G,K)** are from one representative experiment of three independent experiments (mean ± SD, *n* = 3). *p < 0.05; **p < 0.01; ***p < 0.001; n.s. not significant; two-tailed Student’s t-test.

We next explored the molecular basis of KIZ in regulating NF-κB signaling. First, we performed NF-κB luciferase reporter assays in HEK293T cells to screen the target proteins of KIZ in regulating the NF-κB signaling pathway. The reporter assays demonstrated that KIZ overexpression markedly reduced NF-κB activation by overexpression of MyD88, TRAF6, or IRAK1 in a dose-dependent manner (Figures 4A–C), but not by overexpression of TAK1/TAB1, TAB2, or IKKα (Supplementary Figure S4A). These results suggest that KIZ acts upstream of TAK1-TABs complexes to negatively modulate NF-κB signaling.

Next, we conducted co-immunoprecipitation (Co-IP) assays to further identify the target proteins of KIZ by screening KIZ-associated proteins in the NF-κB signaling pathway. HEK293T cells were co-transfected with KIZ and the important components of the NF-κB signaling pathway, which included IRAK1, TRAF6, TAK1, TAB1, TAB2, IKKα, IKKβ, and p65. Co-IP indicated that KIZ strongly associated with TRAF6 and TAK1, but not other proteins (Supplementary Figure S4B). The strong interaction between KIZ and TRAF6 or TAK1 was verified by reverse Co-IP (Supplementary Figure S4C). To confirm the association between KIZ and TRAF6 or TAK1, we performed semi-Co-IP to determinate whether ectopic expression of Flag-tagged KIZ interacted with endogenous TRAF6 and TAK1 with or without IL-1β stimulation. As shown in Figure 4D, we detected IL-1β- and time-dependent associations of KIZ with endogenous TRAF6, but no interaction was detected between KIZ and endogenous TAK1 regardless of IL-1β stimulation. These findings suggested that IL-1β stimulation induced the specific association between KIZ and TRAF6. Additionally, *in vitro* IP assays indicated that KIZ directly interacted with TRAF6, but not GFP (Supplementary Figure S4D). These results suggest that KIZ probably targets TRAF6 to regulate IL-1β-induced NF-κB activation.

To validate this idea, KIZ and TRAF6 were simultaneously knocked down in HeLa cells that were then stimulated with IL-1β, followed by qPCR and immunoblotting. qPCR showed that knockdown of KIZ lost the ability to increase *IL8* and *TNFα* mRNAs induced by IL-1β in TRAF6-knockdown cells (Figures 4E–G). Immunoblotting consistently showed that KIZ knockdown did not increase the levels of phosphorylated TAK1 in TRAF6-knockdown cells (Figure 4H). Similar results were also obtained when TRAF6 was knocked down in *KIZ* ^*−/−*^ HEK293 C6 cells (Supplementary Figures S4E, F). Collectively, these results suggest that KIZ regulates IL-1β-induced NF-κB activation by targeting TRAF6.

Next, we determined which domains of KIZ and TRAF6 were required for their interaction. TRAF6 contains two major domains, an N-terminal zinc-binding domain (TRAF6-N), which is further divided into a RING finger domain and a Zn Finger domain, and a C-terminal TRAF domain (TRAF6-C), which facilitates TRAF protein oligomerization and interactions with other signaling proteins (Kobayashi et al., 2001; Yin et al., 2009; Hu et al., 2017). Thus, we generated two truncated variants of TRAF6, TRAF6-N (only including an N-terminal zinc-binding domain; residues 1–261) and TRAF6-C (only including the C-terminal TRAF domain; residues 262–522). Domain mapping showed that the C-terminal TRAF domain of TRAF6 was required for its interaction with KIZ (Figure 4I). Because KIZ has no reported functional domain, we divided KIZ into three fragments and generated three truncated variants, KIZ-N (residues 1–220), KIZ-Center (residues 221–440), and KIZ-C (residues 441–674). Co-IP indicated that KIZ-N and KIZ-C associated with TRAF6, but the interaction was markedly weaker than that with full length KIZ (Figure 4J). No interaction was detected between KIZ-Center and TRAF6. These results indicate that both the N-terminal and C-terminal of KIZ play important roles in the association between KIZ and TRAF6.

Next, we determined whether the interaction between KIZ and TRAF6 is required for the function of KIZ in regulating NF-κB signaling. Reporter assays showed that KIZ-N and KIZ-C truncated variants had an attenuated function in inhibiting the NF-κB activity induced by TRAF6 compared with full length KIZ (Figure 4K), whereas KIZ-Center had no inhibiting effect. These data suggest that the association between KIZ-TRAF6 plays an important role in the function of KIZ in regulating NF-κB signaling.

### KIZ Targets TRAF2 to Modulate NF-ĸB Activation Triggered by TNFα

**FIGURE 5:**
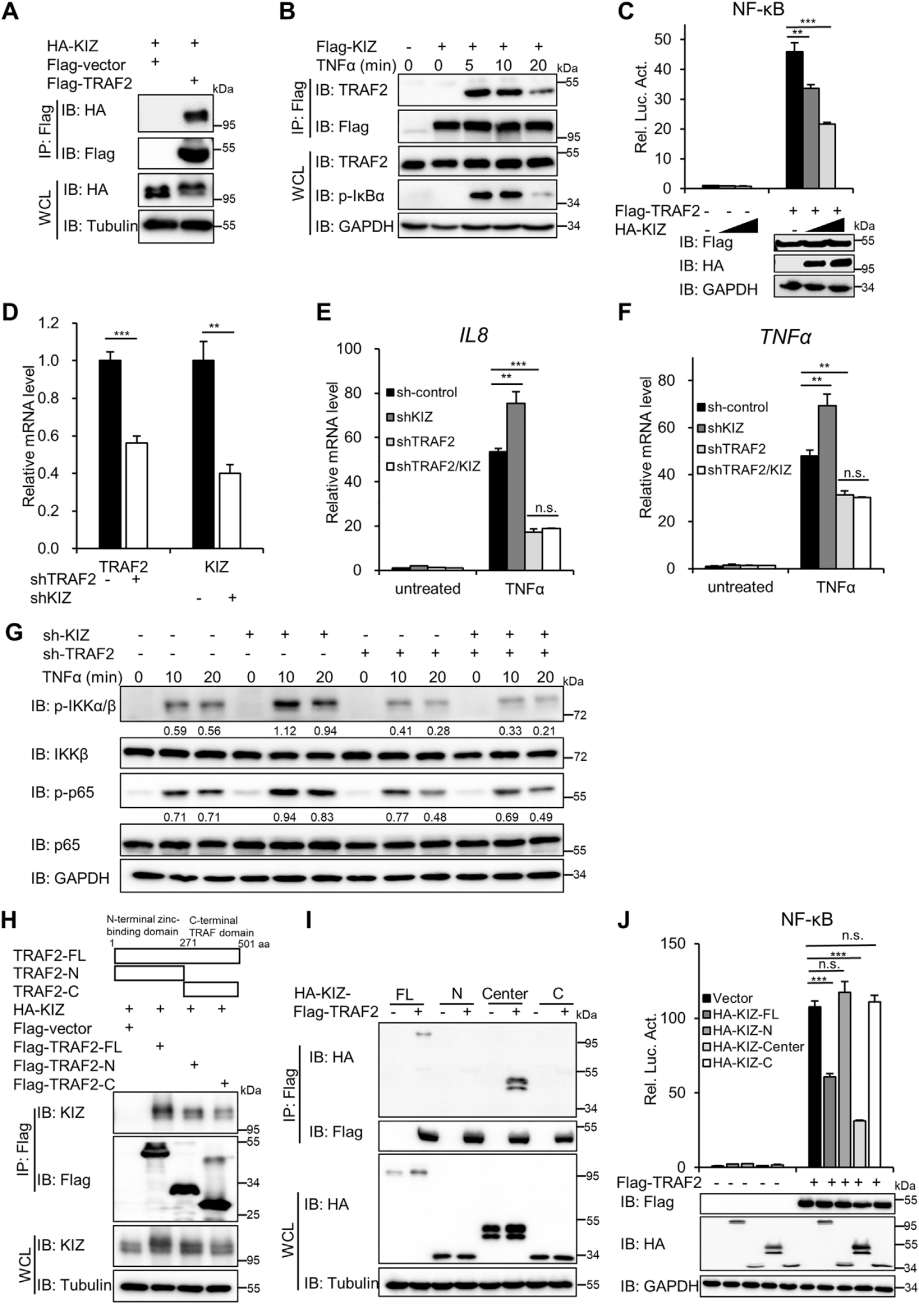
KIZ regulates TNFα-induced NF-ĸB activation by targeting TRAF2. **(A)** HEK293T cells were transfected with the indicated expression plasmids. At 24 h after transfection, cell lysates were subjected to Co-IP with anti-Flag beads and analyzed by immunoblotting. **(B)** HEK293T cells were transfected with a Flag-tagged KIZ expression plasmid or an empty vector. After 24 h, the cells were stimulated with TNFα (20 ng/ml) for the indicated times and then lysed for Co-IP with anti-Flag beads, followed by immunoblotting. **(C)** HEK293T cells were transfected with increasing doses of KIZ plasmids or an empty vector together with a NF-κB-Luc reporter and plasmids that expressed Flag-TRAF2 or an empty vector. After 24 h of transfection, the cells were lysed for luciferase reporter assays (upper panel) and immunoblotting (lower panels). **(D–G)** U2OS cells were infected with an shRNA lentivirus that targeted KIZ, TRAF2, both, or the empty vector for 48 h. The cells were untreated or treated with TNFα (20 ng/ml) for 3 h and then analyzed by qPCR to measure the mRNA levels of *KIZ*, *TRAF2* **(D)**, *IL8* **(E)**, and *TNFα* **(F)** or treated for the indicated times, followed by immunoblotting **(G)**. **(H)** Schematic diagram of TRAF2 domains (upper panel). HEK293T cells were co-transfected with KIZ and TRAF2 or its truncated variants. After 24 h, the cells were lysed for IP with anti-Flag beads and analyzed by immunoblotting (lower panels). **(I)** HEK293T cells were co-transfected with TRAF2 and KIZ or its truncated variants. After 24 h, the cells were lysed for Co-IP with anti-Flag beads and analyzed by immunoblotting. **(J)** HEK293T cells were transfected with Flag-TRAF2 or an empty vector together with an NF-κB-Luc reporter and the plasmids that expressed HA-tagged KIZ or its variants. After 24 h of transfection, the cells were lysed for luciferase reporter assays (upper panel) and immunoblotting (lower panels). Data shown in **(C–F,J)** are from one representative experiment of three independent experiments (mean ± SD, *n* = 3). **p < 0.01; ***p < 0.001; n.s. not significant; two-tailed Student’s t-test.

Next, we investigated how KIZ modulated TNFα-induced NF-κB signaling. Considering that TRAF2 plays an important role in the activation of NF-κB induced by TNFα and shares similar structures to TRAF6, we examined whether KIZ interacts with TRAF2. Co-IP indicated that KIZ had a strong association with TRAF2 (Figure 5A). Next, to examine whether TNFα stimulation affected the association between TRAF2 and KIZ, we conducted semi-Co-IP and observed that ectopic KIZ pulled down endogenous TRAF2 only in the presence of TNFα, which was also dependent on time (Figure 5B). These results suggest that KIZ negatively modulates TNFα-induced NF-κB signaling probably by targeting TRAF2.

To test this hypothesis, we performed NF-κB reporter assays and found that KIZ overexpression attenuated TRAF2-induced NF-κB activity (Figure 5C). Next, we conduced TRAF2 and KIZ double knockdown experiments. qPCR showed that knockdown of KIZ did not enhance the mRNA levels of *IL8* and *TNF*α induced by TNFα in TRAF2 knockdown cells (Figures 5D–F). Moreover, immunoblotting showed that KIZ knockdown did not augment the levels of phosphorylated IKKα/β and p65 in TRAF2 knockdown cells (Figure 5G). These results suggest that KIZ reduces NF-κB activity induced by TNFα by targeting TRAF2.

Next, we conducted domain mapping experiments to determinate which domains of KIZ and TRAF2 are required for their interaction. Similar to TRAF6, TRAF2 also contains two major domains, an N-terminal zinc-binding domain (TRAF2-N) and a C-terminal TRAF domain (TRAF2-C) (Rothe et al., 1995; Takeuchi et al., 1996). Therefore, we generated two truncated variants of TRAF2, TRAF2-N (only including an N-terminal zinc-binding domain; residues 1–271) and TRAF2-C (only including C-terminal TRAF domain; residues 272–501). Co-IP showed that both the N-terminal zinc-binding domain and TRAF domain of TRAF2 interacted with KIZ, but the interactions were much weaker than that with full length TRAF2 (Figure 5H). These data suggested that both the N-terminal zinc-binding domain and TRAF domain of TRAF2 were involved in the association of KIZ with TRAF2. Interestingly, through protein sequence analysis, we identified residues 244–247 and 416–419 of KIZ as potential TRAF2-binding motifs, which harbored a consensus (P/S/A/T)-X-(Q/E)-E sequence (Ye et al., 1999). Co-IP showed that the KIZ-Center fragment (residues 221–440), but not KIZ-N or KIZ-C, associated with TRAF2, which suggested that the center fragment of KIZ played an important role in the association between KIZ and TRAF2 (Figure 5I). Next, we examined whether the association between KIZ and TRAF2 was required for the function of KIZ in TRAF2-mediated signaling. Reporter assays showed that, similar to full length KIZ, the KIZ-Center fragment decreased NF-κB activity triggered by TRAF2, whereas KIZ-N and KIZ-C fragments had no inhibiting effects (Figure 5J). These data demonstrate that the interaction between KIZ and TRAF2 is required for KIZ’s function in mediating NF-κB signaling induced by TRAF2.

### KIZ Antagonizes the Association of TRAF2/TRAF6 With Upstream Adaptors

Next, we explored how KIZ decreased NF-κB activation induced by IL-1β *via* targeting TRAF6. Because TRAF6 functions as E3 ligase to catalyze K63-linked polyubiquitination chains, subsequently activates the TAK1 and IKK complexes, we examined whether KIZ regulated the synthesis of K63-linked ubiquitination chains catalyzed by TRAF6. *In vitro* ubiquitination assays showed that KIZ had no effects on the synthesis of K63-linked ubiquitination chains catalyzed by TRAF6 (Supplementary Figure S5). Previous studies showed that in addition to its function as E3 ubiquitin ligase, TRAF6 also acts as an adaptor protein to link upstream receptors to downstream effectors through its TRAF domain (H. H. Park 2018; Xie 2013b; Inoue et al., 2000; Wajant et al., 2001; Ha et al., 2009). Combined with the results showed above that KIZ acted upstream of TAK1 complex and interacted with TRAF6 through TRAF domain, thus, we speculated that KIZ probably affected the association of TRAF6 with upstream adaptors to decrease NF-ĸB signaling induced by IL-1β.

**FIGURE 6:**
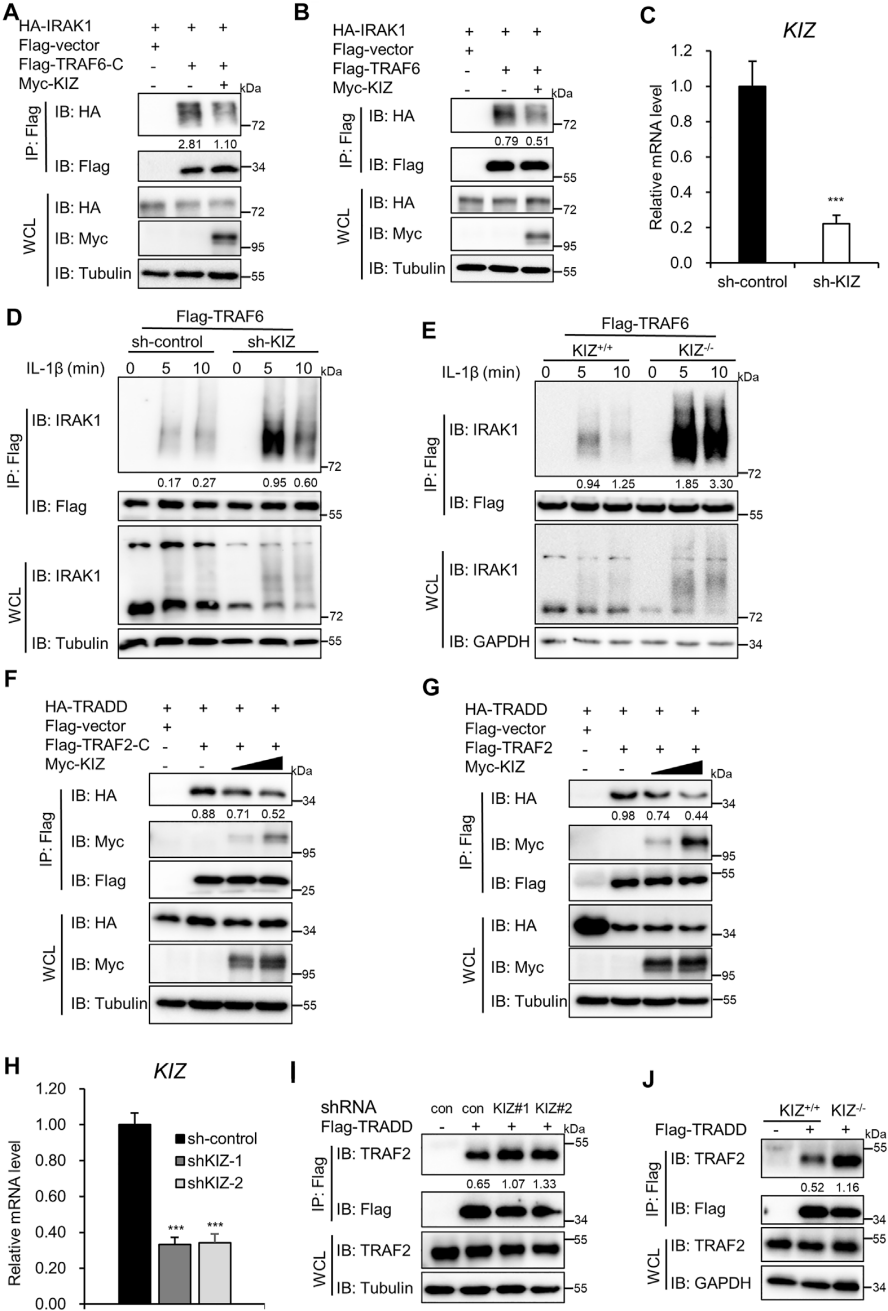
KIZ antagonizes the association of TRAF2/TRAF6 with upstream adaptors. **(A,B)** HEK293T cells were co-transfected with the indicated plasmids. Cell lysates were immunoprecipitated with anti-Flag beads, followed by immunoblotting. **(C,D)** HEK293 C6 cells were infected with an shRNA lentivirus that targeted *KIZ* or the empty vector for 48 h and then the cells were transfected with a Flag-tagged TRAF6 expression plasmid. After 24 h of transfection, the cells were untreated or treated with IL-1β (5 ng/ml) for the indicated times and then lysed for qPCR to examine KIZ knockdown efficiency **(C)** or for Co-IP with anti-Flag beads, followed by immunoblotting **(D)**. **(E)** WT and KIZ knockout HEK293 C6 cells were transfected with a Flag-tagged TRAF6 expression plasmid. After 24 h of transfection, the cells were untreated or treated with IL-1β (5 ng/ml) for the indicated times and then lysed for Co-IP with anti-Flag beads, followed by immunoblotting. **(F,G)** HEK293T cells were co-transfected with the indicated plasmids. Cell lysates were immunoprecipitated with anti-Flag beads and analyzed by immunoblotting. **(H,I)** HEK293T cells were infected with two shRNA lentivirus that targeted KIZ or an empty vector for 48 h and then the cells were transfected with a Flag-tagged TRADD expression plasmid or an empty vector. After 24 h of transfection, the cells were lysed for qPCR to examine KIZ knockdown efficiency **(H)** or for Co-IP with anti-Flag beads, followed by immunoblotting **(I)**. **(J)** WT and KIZ knockout HEK293 C6 cells were transfected with a Flag-tagged TRADD expression plasmid or an empty vector. After 24 h of transfection, the cells were lysed for Co-IP with anti-Flag beads, followed by immunoblotting. Data shown in **(C,H)** are from one representative experiment of three independent experiments (mean ± SD, *n* = 3). ***p < 0.001; two-tailed Student’s t-test.

IRAK1 acts upstream of TRAF6 in the activation of NF-ĸB induced by IL-1β. Interestingly, IRAK1 interacts with TRAF6 through the C-terminal TRAF domain of TRAF6 (Cao et al., 1996). Our above domain mapping experiments also showed that the association between KIZ and TRAF6 relied on the C-terminal TRAF domain of TRAF6 (Figure 4I). Thus, we determined whether KIZ competes with IRAK1 to interact with the C-terminal TRAF domain of TRAF6. In line with this, we found that IRAK1 interacted with the C-terminal TRAF domain of TRAF6, and importantly, KIZ overexpression reduced their interaction (Figure 6A). We next examined whether KIZ antagonizes the association of TRAF6 with IRAK1. The Co-IP results in Figure 6B indicated that ectopic expression of KIZ attenuated the association of IRAK1 with TRAF6. Next, we investigated whether depletion of KIZ affected the interaction between IRAK1 and TRAF6. Co-IP showed that, following IL-1β stimulation, the association of IRAK1 with TRAF6 was dramatically potentiated in KIZ knockdown (Figures 6C,D) or knockout (Figure 6E) HEK293 C6 cells. These data suggest that KIZ preferentially binds to the C-terminal TRAF domain of TRAF6, which decreases the association between TRAF6 and IRAK1.

Next, we explored how KIZ regulated TNFα-induced NF-κB signaling by targeting TRAF2. Considering that the structure and function of TRAF2 are very similar to TRAF6, we hypothesized that KIZ probably mediated TNFα-induced NF-κB signaling by antagonizing the association of TRAF2 with the upstream adaptor. Considering that TRADD acts upstream of TRAF2 in TNFα-induced NF-κB signaling, the C-terminal TRAF domain of TRAF2 was required for the interaction with TRADD (Hsu et al., 1996b), and our above domain mapping results also showed that KIZ interacted with the C-terminal TRAF domain of TRAF2 (Figure 5H). Thus, we examined whether KIZ competed with TRADD to interact with the C-terminal TRAF domain of TRAF2. Co-IP results showed that TRADD interacted with the C-terminal TRAF domain of TRAF2 and KIZ overexpression attenuated their association (Figure 6F). Next, we investigated whether KIZ affects the interaction of TRADD with TRAF2. As shown in Figure 6G, KIZ overexpression remarkedly decreased the association between overexpressed TRADD and TRAF2 in a dose-dependent manner. Conversely, the interaction of TRADD with TRAF2 was augmented in KIZ knockdown or knockout cells (Figures 6H–J). Taken together, these results suggest that KIZ antagonizes the association of TRADD with TRAF2 to maintain the balance of NF-κB signaling induced by TNFα.

### GM114 Attenuates DSS-Induced Acute Colitis

**FIGURE 7:**
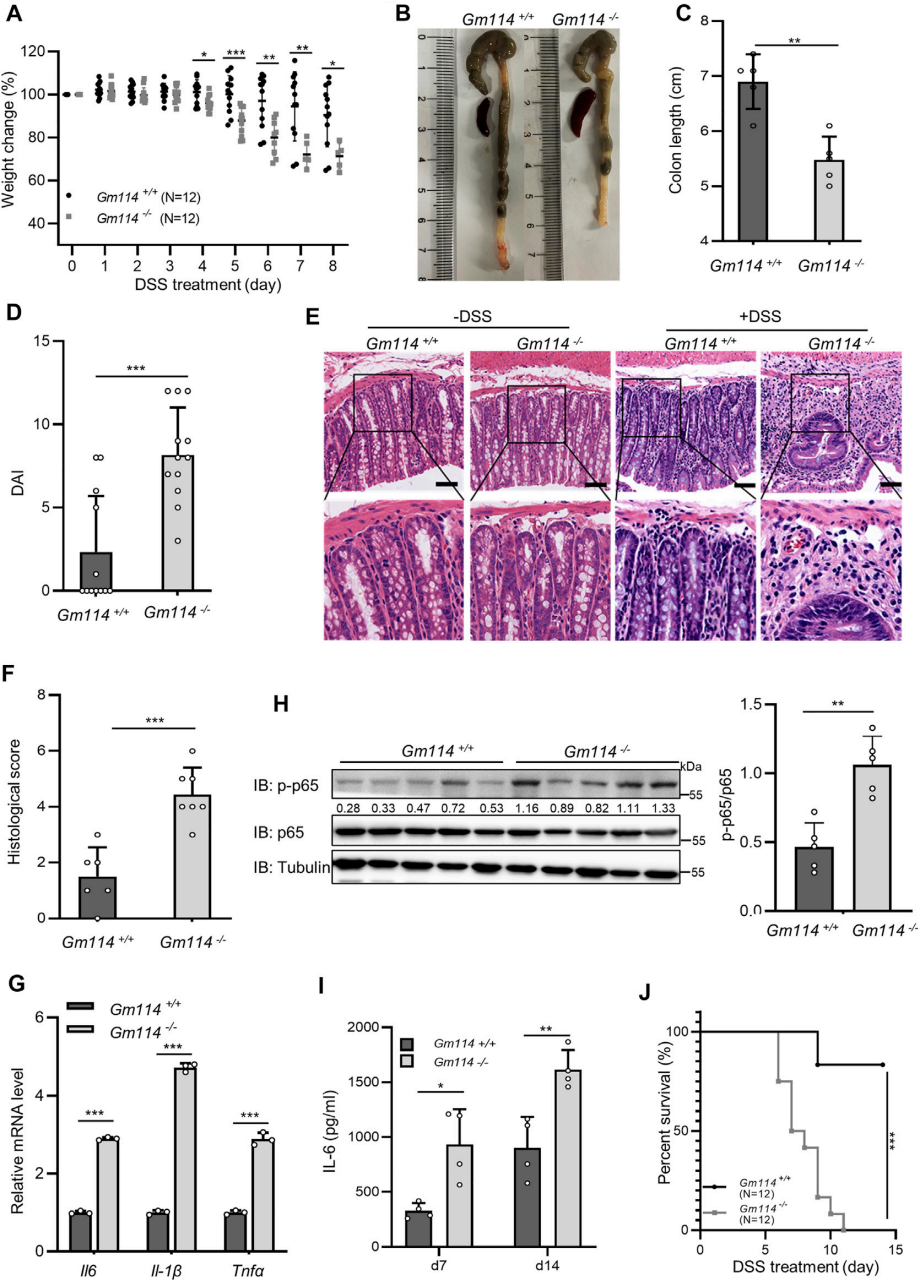
GM114 attenuates DSS-induced acute colitis. **(A)** *Gm114* ^+/+^ and *Gm114* ^*−/−*^ mice were treated with 5% (w/v) DSS in drinking water for 5 days, followed by 9 days of normal drinking water. Body weight changes were recorded daily until the mice had mostly died (day 8). Data represent the weight change relative to each individual starting weight. **(B,C)** *Gm114* ^+/+^ and *Gm114* ^*−/−*^ mice were treated with 2.5% (w/v) DSS in drinking water for 7 days, followed by 7 days of normal drinking water. Mice were sacrificed on day 14 to measure the colon length. **(D)** Mice were treated as described in **(A)**. Composite DAI scores were determined by assessing weight loss, stool consistency, and the presence of blood in stool and/or the rectum at day 5 after DSS treatment. **(E,F)** Mice were treated as described in **(B)**. Representative H&E-stained sections of colons from mock- and DSS-treated *Gm114* ^+/+^ and *Gm114* ^*−/−*^ mice (14 days). Scale bar represents 50 μm **(E)**. Histological scores of inflammation-associated changes in the colon were assessed as shown in **(F)**. **(G)** Mice were treated as described in **(B)**. qPCR was conducted to measure the mRNA levels of *Il6*, *Il-1*β and *Tnfα* in colons from *Gm114* ^+/+^ and *Gm114* ^*−/−*^ mice at day 14 after 2.5% DSS administration. **(H)** Mice were treated as described in **(B)**. Colons from Gm114^+/+^ and Gm114^−/−^ mice at day 14 after 2.5% DSS administration were harvested for immunoblotting (left panel), and densitometric analysis was shown in right panel. **(I)** Mice were treated as described in **(B)**. Sera were collected at days 7 and 14 after DSS administration to measure IL-6 production by an ELISA. **(J)** Mice were treated as described in **(A)**. Survival rates of *Gm114* ^+/+^ and *Gm114* ^*−/−*^ mice were monitored daily. Data shown in **(A,C,D,F,G,I,J)** are from one representative experiment of three independent experiments [mean ± SD, *n* = 3 in **(G)**]. *p < 0.05; **p < 0.01; ***p < 0.001; two-tailed Student’s t-test. The log-rank (Mantel–Cox) test was used in Data **(J)**.

NF-κB signaling has an important role in regulating the inflammatory response. Our functional assays demonstrated that KIZ/GM114 negatively modulated NF-κB signaling induced by IL-1β/LPS and TNFα. Next, we examined whether GM114 exerted a protective effect on regulating the inflammatory response *in vivo*. To test this hypothesis, we used a mouse model of colitis induced by dextran sulfate sodium (DSS), which is a widely employed model of inflammatory bowel disease with many similar features to human inflammatory bowel diseases (Okayasu et al., 1990). NF-κB mediated the production of various proinflammatory cytokines, such as IL-6, TNFα, and IL-1β, which regulated inflammatory responses in colitis induced by DSS (M. L. Chen and Sundrud 2016; Neurath 2014; Fantini and Pallone 2008). As shown in Figures 7A–F, *Gm114* knockout mice exhibited higher colon inflammation that included a faster progressive body weight loss (Figure 7A), a shorter colon length (Figures 7B,C), and an enhanced disease activity index (DAI) (Figure 7D) as wells as more inflammatory cell infiltration and disrupted mucosal structures in histological analyses of the colons by H&E staining (Figures 7E,F). Next, we performed qPCR assays to measure the levels of proinflammatory cytokines and found that the mRNA levels of *Il6*, *Il1β* and *Tnfα* induced by DSS treatment were significantly higher in the colons of *Gm114* ^−/−^ mice than those in *Gm114* ^+/+^ mice (Figure 7G). Consistently, immunoblotting results showed increased levels of phosphorylated p65 in the colons of *Gm114* knockout mice compared with those of control mice (Figure 7H). Moreover, the results of ELISA also suggested that *Gm114* knockout significantly augmented serum IL-6 (Figure 7I). In addition, *Gm114* knockout mice had a lower survival rate upon DSS treatment (Figure 7J). Taken together, these results demonstrate that GM114 suppresses DSS-induced acute colitis in mice by downregulating the inflammatory response.

## Discussion

NF-κB signaling plays an important role in regulating the inflammatory responses by inducing the expression of proinflammatory genes, including cytokines, chemokines, and adhesion molecules (Popa et al., 2007; Lawrence 2009). Unrestrained NF-κB activation has been linked to many diseases such as inflammatory and autoimmune diseases, and even cancer (Cook et al., 2004; Marshak-Rothstein 2006; Simmonds and Foxwell 2008; Landskron et al., 2014). However, how NF-κB activation is tightly controlled to maintain inflammatory homeostasis remains unclear. In this study, we identified KIZ/GM114 as a negative modulator of NF-κB signaling induced by IL-1β/LPS and TNFα. We demonstrated that KIZ bound to TRAF6/2 to antagonize the association of TRAF6 with IRAK1 or TRAF2 with TRADD in NF-κB signaling induced by IL-1β/LPS or TNFα, respectively (Supplementary Figure S6). Furthermore, we found that GM114 reduced DSS-induced acute colitis in mice by attenuating the inflammatory responses. These data suggest that KIZ/GM114 plays a negative role in regulating NF-κB signaling to maintain inflammatory homeostasis.

Our previous study demonstrated that *Drosophila* Bam played an important role in regulating IMD signaling by targeting TRAF6 to regulate the synthesis of K63-linked ubiquitination chains (Ji et al., 2019). In this study, we found that KIZ/GM114 also negatively modulated NF-κB signaling induced by IL-1β/LPS and TNFα. Interestingly, KIZ/GM114 did not affect the E3 ligase activity of TRAF6, but affected the association of TRAF6 with IRAK1 or TRAF2 with TRADD, respectively. These results suggest that KIZ/GM114 negatively regulates NF-κB signaling via a different mechanism from Bam. Additionally, considering that Bam is involved in regulating the *Drosophila* lifespan by affecting intestinal immune homeostasis and that GM114 reduced DSS-induced acute colitis in mice by attenuating the inflammatory responses, it would be of great interest to study whether KIZ/GM114 plays a conserved role in lifespan in the future.

In addition to their role as E3 ubiquitin ligases, TRAF2 and TRAF6 also function as adaptor proteins and play important roles in the assembly of receptor-associated signaling complexes by linking upstream receptors to downstream effectors through the C-terminal TRAF domain (H. H. Park 2018; Xie 2013a; Wajant et al., 2001). In this study, we found that KIZ interacted with the TRAF domain of TRAF2 and TRAF6, and subsequently antagonized the association of TRAF2/6 with their upstream adaptor proteins. Moreover, we found that KIZ interacted with TRAF6 and TRAF2 in a ligand-dependent manner. These results suggest that KIZ functions as a new inducible negative factor in modulation of NF-κB signaling.

Considering that NF-κB signaling is involved in regulating many biological processes, such as innate immunity, the inflammatory responses, cell survival and cell death, inappropriate NF-κB signaling may contribute to many human diseases, such as rheumatoid arthritis, infectious and inflammatory diseases, and even cancers. Therefore, our finding provides new insights into KIZ as a therapeutic target for the treatment of related diseases.

## Materials and Methods

### Ethics Statement

All animal studies were performed in accordance with the recommendations in the Guide for the Care and Use of Laboratory Animals of the Ministry of Science and Technology of the People’s Republic of China. The protocols for animal studies were approved by the Committee on the Ethics of Animal Experiments of the Institute of Zoology, Chinese Academy of Sciences (Approval number: IOZ15001).

### Cell Culture and Animals

HEK293T, HeLa, RAW264.7 cells and U2OS cells were cultured with high-glucose DMEM (Gibco) medium containing 10% heat-inactivated fetal bovine serum (Invitrogen) and 1% streptomycin and penicillin (Gibco). THP-1 cells were cultured in RPMI-1640 containing 10% fetal bovine serum, 1% streptomycin and penicillin, 10 mM HEPES (pH7.0), and 10 µM β-mercaptoethanol. *Gm114* ^*+/+*^ and *Gm114* ^*−/−*^ MEFs were generated from 13.5-day-old embryos and maintained in complete DMEM containing 1 mM sodium pyruvate (Gibco), 10 μM L-glutamine, 10 μM β-mercaptoethanol, and 1% nonessential amino acids (Gibco). Bone marrow cells were isolated from 8-weeks-old *Gm114* ^*+/+*^ and *Gm114* ^*−/−*^ mice and cultured with IMDM (Gibco) medium containing conditioned media from L929 cell culture, 10% fetal bovine serum, 1 mM sodium pyruvate and 1% streptomycin and penicillin for 5 days to generate BM-derived macrophages (BMDM).

### Antibodies

Rabbit anti-p-p65 (Ser536, 3033), anti-p-TAK1 (T184/187,4508), anti-TAK1 (5206), anti-p-IKKα/β (S176/180, 2697), anti-IKKβ (8943), anti-IRAK1 (4504), anti-IκBα (9242) and mouse anti-p-IκBα (Ser32/36, 9246) antibodies were purchased from Cell Signaling Technology. Rabbit anti-TRAF2 (SC-876) and mouse anti-p65 (SC-8008), anti-ubiquitin (SC-8017) antibodies were purchased from Santa Cruz Biotechnology. Rabbit anti-TRAF6 (ab40675) was purchased from Abcam. Other antibodies used were as follows: Rabbit anti-Flag (Sigma); rabbit anti-HA (MBL); rabbit anti-Myc (MBL); mouse anti-GAPDH (Sungene Biotechnology, KM9002) and mouse anti-αTubulin (Sungene Biotechnology, KM9007). The antibodies against KIZ and GM114 were generated by immunizing mice with the recombinant proteins His_6_-KIZ (residues 563-674) and His_6_-GM114 (residues 149-294) produced in *E. coli*, respectively.

### Plasmids

Human full length KIZ and mouse full length GM114 were cloned into pEF vectors. KIZ truncated variants were cloned into pEF-HA vectors. The plasmids encoding MyD88, TRAF6, TAK1, TAB1, TAB2, IKKα, IKKβ, p65, Renilla reporter, and NF-κB-Luc reporter have been described previously (E. Liu et al., 2021).

### Lentivirus-Mediated KIZ and GM114 Overexpression and shRNA-Mediated Knockdown

Full length cDNA that encoded KIZ or Gm114 was amplified and inserted into a pCDH-CMV-Puro vector. Lentivirus particles for KIZ or GM114 overexpression were produced by co-transfecting the pCDH-CMV-Puro-KIZ or GM114 construct into HEK293T cells with the packaging plasmids pMD2.G and pAS-MAX. To generate knockdown cells, we employed pLKO.1-puro-based lentiviruses that expressed specific short hairpin RNAs (shRNAs) against KIZ, TRAF6 or TRAF2. Cells were infected with shRNA lentiviruses that targeted the indicated gene or a control vector (pLKO.1). The knockdown efficiency was determined by qPCR. The shRNA sequences against the KIZ, TRAF6 and TRAF2 were as follows (5′–3′):

shKIZ-1: GTC​TGA​TAC​ATG​CAG​AGT​TAA;
shKIZ-2: CAA​GGG​AAC​AAG​AAG​TTT​CAA;
shTRAF6: GCC​ACG​GGA​AAT​ATG​TAA​TAT;
shTRAF2: CTC​GGG​CAT​GAC​AGG​CAG​AAA.

### CRISPR/Cas9-Mediated KIZ Knockout Cell Lines

Two sgRNAs that targeted different DNA regions of *KIZ* were inserted into the pX330-GFP-U6-Chimeric_BB-CBh-hSpCas9 vector. HEK293 C6 cells were co-transfected with these two expression plasmids. Twenty-four hours later, single cells that expressed GFP were isolated by fluorescence-activated cell sorting. Knockout cells were verified by immunoblotting. The sgRNA sequences were (5′→3′):

KIZ-gRNA#1: CGG​CCC​ATA​AAG​ATT​GTT​GC TGG;
KIZ-gRNA#2: ACT​TAA​TCT​TTC​CCG​TAA​CG GGG.

### Transfection and Luciferase Reporter Assay

HEK293T cells were transfected with various expression plasmids along with NF-κB-Luc (10 ng/well) and Renilla reporter plasmid (25 ng/well) in a 24-well plate using polyethylenimine. Twenty-four hours post-transfection, the cells were lysed using passive lysis buffer (Promega) for luciferase activity, followed by immunoblotting, and transfection efficiency was normalized to Renilla activity.

### Co-IP and Immunoblotting

For *in vivo* Co-IP, cells were lysed in lysis buffer (0.5% Triton X-100, 20 mM Tris-HCl, pH 7.5, 150 mM NaCl, 10% glycerol, and 1 mM EDTA) with a fresh protease inhibitor cocktail (Roche). Clarified supernatants were incubated with anti-Flag agarose beads (Sigma) or anti-HA magnetic beads (Pierce) for 4 h at 4°C. Immunoprecipitated complexes were washed three times with lysis buffer that contained 300 mM NaCl and then subjected to immunoblotting with the indicated antibodies.

For *in vitro* pull-down experiments, recombinant GFP-TRAF6 protein, GFP, and His-KIZ were purified from *E. coli*. His-KIZ protein was incubated with GFP or GFP-TRAF6 at 4°C for 3 h and then incubated with anti-GFP agarose beads at 4°C for 2 h, washed three times with washing buffer (25 mM Tris-HCl pH 7.5, 300 mM NaCl, 10% glycerol) and subjected to immunoblotting.

### *In vitro* Ubiquitination Assay

Recombinant E1, Ubc13/Uev2, and ubiquitin proteins were kindly provided by Dr Zongping Xia (Zhengzhou University). His-TRAF6 and His-KIZ recombinant proteins were purified from *E. coli.* For *in vitro* ubiquitination assays, recombinant E1, Ubc13/Uev2, ubiquitin, and His-TRAF6 were mixed in ATP buffer (50 mM Tris-HCl, pH 7.5, 5 mM MgCl_2_, 2 mM ATP, and 0.5 mM DTT) in the presence or absence of His-KIZ recombinant protein. The reaction mixture was incubated for 1 h at 30°C and the reaction was terminated by addition of denaturing sample buffer, followed by heating at 95°C for 5 min. The samples were resolved on 6–18% or 10% SDS-PAGE gels, followed by immunoblotting.

### qPCR

Total RNA was extracted using TRIzol reagent (Invitrogen) in accordance with the manufacturer’s instructions. cDNA was synthesized using a HiScript III 1st Strand cDNA Synthesis Kit (Vazyme). Quantitative PCR was performed in triplicate using SYBR Green Master Mix (Thermo Fisher) on a Bio-Rad CFX connect system. Relative levels of mRNA were normalized to the levels of GAPDH in each sample. Data shown are relative mRNA abundance compared with control groups.

The primers used for qPCR were as follows (5′-3′):

h*KIZ*-S GGT​GTT​TCA​GAT​CAT​CTT​GCT​C;
h*KIZ*-AS CCT​CTA​TTC​CAT​CTT​CTC​CCT​C;
h*IL6*-S TCC​AGA​ACA​GAT​TTG​AGA​GTA​GTG;
h*IL6*-AS GCA​TTT​GTG​GTT​GGG​TCA​GG;
h*IL8*-S ATA​AAG​ACA​TAC​TCC​AAA​CCT​TTC​CAC;
h*IL8*-AS AAG​CTT​TAC​AAT​AAT​TTC​TGT​GTT​GGC;
h*TNFα*-S CTG​CCC​CAA​TCC​CTT​TAT​T;
h*TNFα*-AS CCC​AAT​TCT​CTT​TTT​GAG​CC;
h*TRAF6*-S TTT​GCT​CTT​ATG​GAT​TGT​CCC​C;
h*TRAF6*-AS CAT​TGA​TGC​AGC​ACA​GTT​GTC;
h*TRAF2*-S TCC​CTG​GAG​TTG​CTA​CAG​C;
h*TRAF2*-AS AGG​CGG​AGC​ACA​GGT​ACT​T;
h*IFIT1*-S TAC​CTG​GAC​AAG​GTG​GAG​AA;
h*IFIT1*-AS GTG​AGG​ACA​TGT​TGG​CTA​GA;
h*GAPDH*-S ATG​ACA​TCA​AGA​AGG​TGG​TG;
h*GAPDH*-AS CAT​ACC​AGG​AAA​TGA​GCT​TG;
m*Il6*-S TCG​GAG​GCT​TAA​TTA​CAC​ATG​TTC​T;
m*Il6*-AS TGC​CAT​TGC​ACA​ACT​CTT​TTC​T;
m*Il-1β*-S AAA​GCC​TCG​TGC​TGT​CGG​ACC;
m*Il-1β*-AS CAG​GGT​GGG​TGT​GCC​GTC​TT;
m*Tnfα*-S TCC​CCA​AAG​GGA​TGA​GAA​GTT;
m*Tnfα*-AS GTT​TGC​TAC​GAC​GTG​GGC​TAC;
m*Gapdh*-S AAC​TTT​GGC​ATT​GTG​GAA​GG;
m*Gapdh*-AS ACA​CAT​TGG​GGG​TAG​GAA​CA.

### Experimental Colitis Model

Mice were administered 2.5% (w/v) DSS (MW = 36,000–50,000; MP Biomedicals, Irvine, CA) in drinking water for a period of 7 days, followed by normal drinking water for 7 days or 5% (w/v) DSS for 5 days, followed by normal drinking water. The survival rate and weight loss of the mice were monitored for 14 consecutive days from the first day of DSS exposure to the day of death. Disease activity index (DAI) scores were evaluated on day 5 after DSS treatment on the basis of weight loss, stool consistency, and the degree of rectum bleeding (Guo et al., 2014). Sera were collected at days 7 and 14 to measure IL-6 by a mouse IL-6 ELISA kit (BD Biosciences), following the manufacturer’s instructions. At day 14 after DSS treatment, mice were sacrificed to measure the colon length, followed by hematoxylin and eosin (H&E) staining of the colon in accordance with standard protocols. On the basis of the H&E staining of a colon section, evaluation of inflammation-associated histological changes in the colon was performed using a scale described previously by Stefan Wirtz et al. (2017). Other colon tissues were lysed for qPCR analysis or immunoblotting.

### Statistical Analysis

The log-rank (Mantel–Cox) test was used for survival rate analysis. Other statistical analyses are shown as mean ± SD. Statistical analysis was carried out using the two-tailed Student’s *t*-test (GraphPad Prism 8.0). For all tests, *p*-values of <0.05 were considered statistically significant.

## Data Availability

The original contributions presented in the study are included in the article/Supplementary Material, further inquiries can be directed to the corresponding authors.
