# Supplementary figures and images for: KIZ/GM114 Balances the NF-ĸB Signaling by Antagonizing the Association of TRAF2/6 With Their Upstream Adaptors
Source: Front Cell Dev Biol. 2022 Mar 31;10:877039. doi: 10.3389/fcell.2022.877039 (PMC9008698; doi:10.3389/fcell.2022.877039)

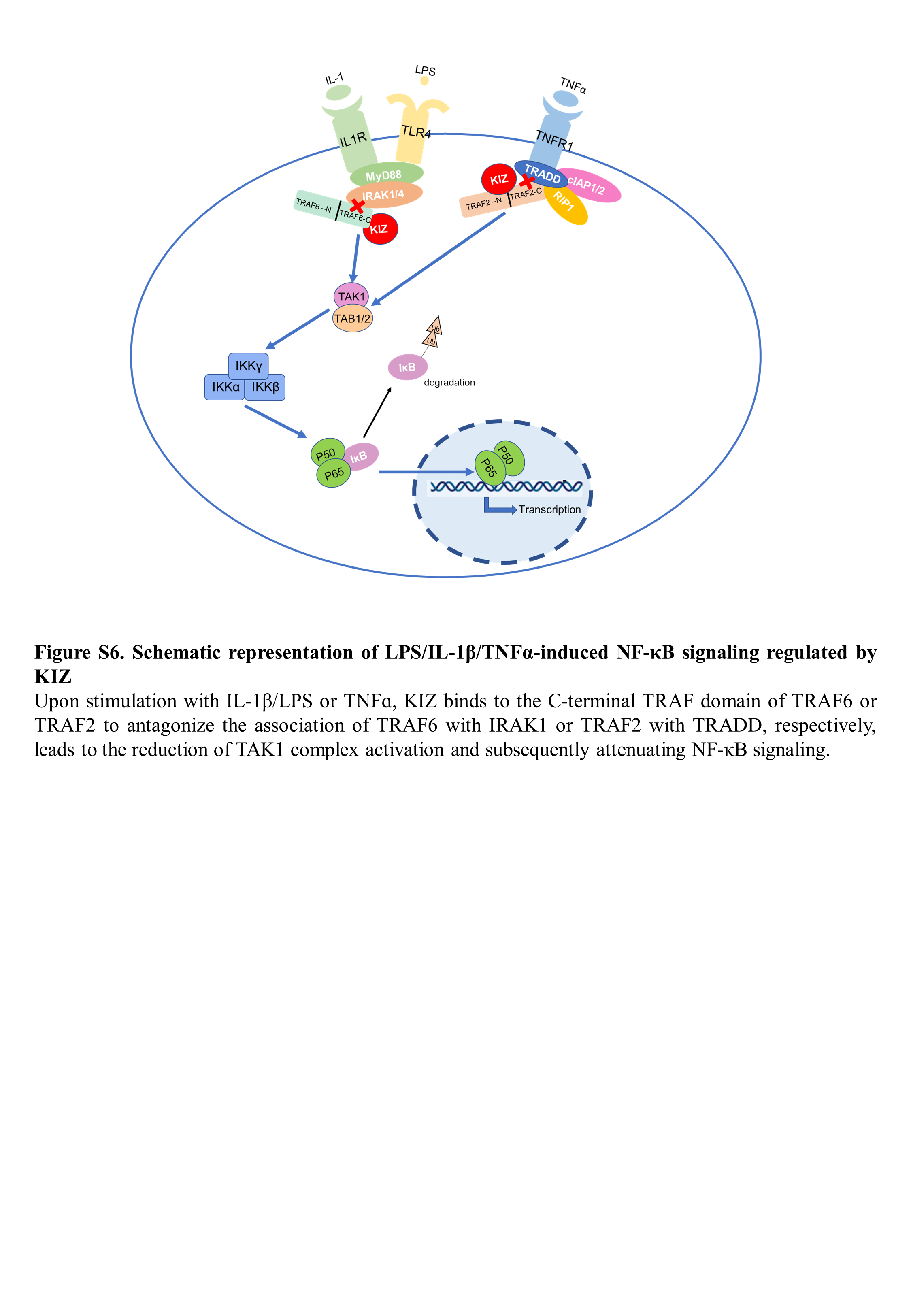

Supplement: Supplementary file 1 [file Image6.TIF]

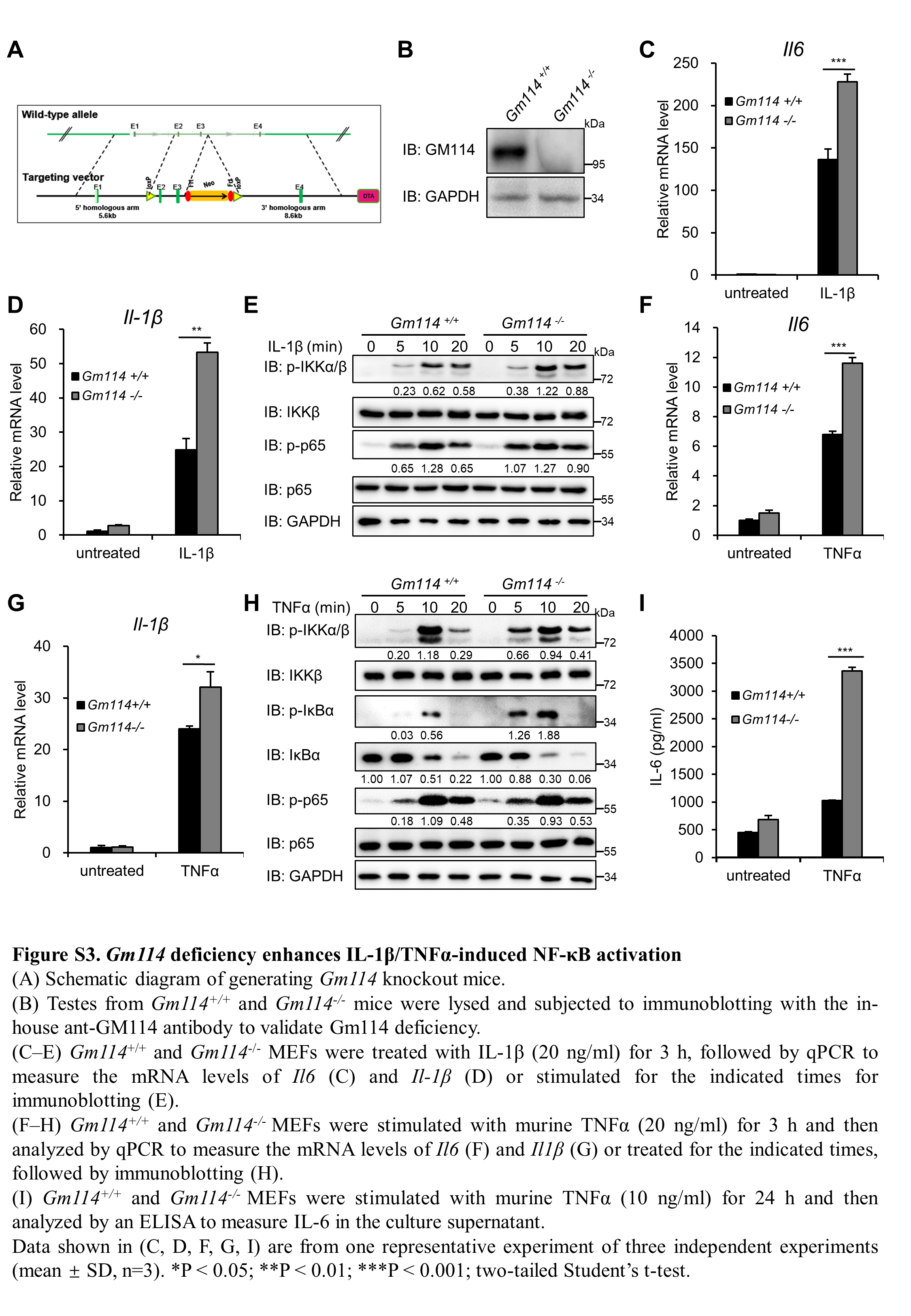

Supplement: Supplementary file 2 [file Image3.TIF]

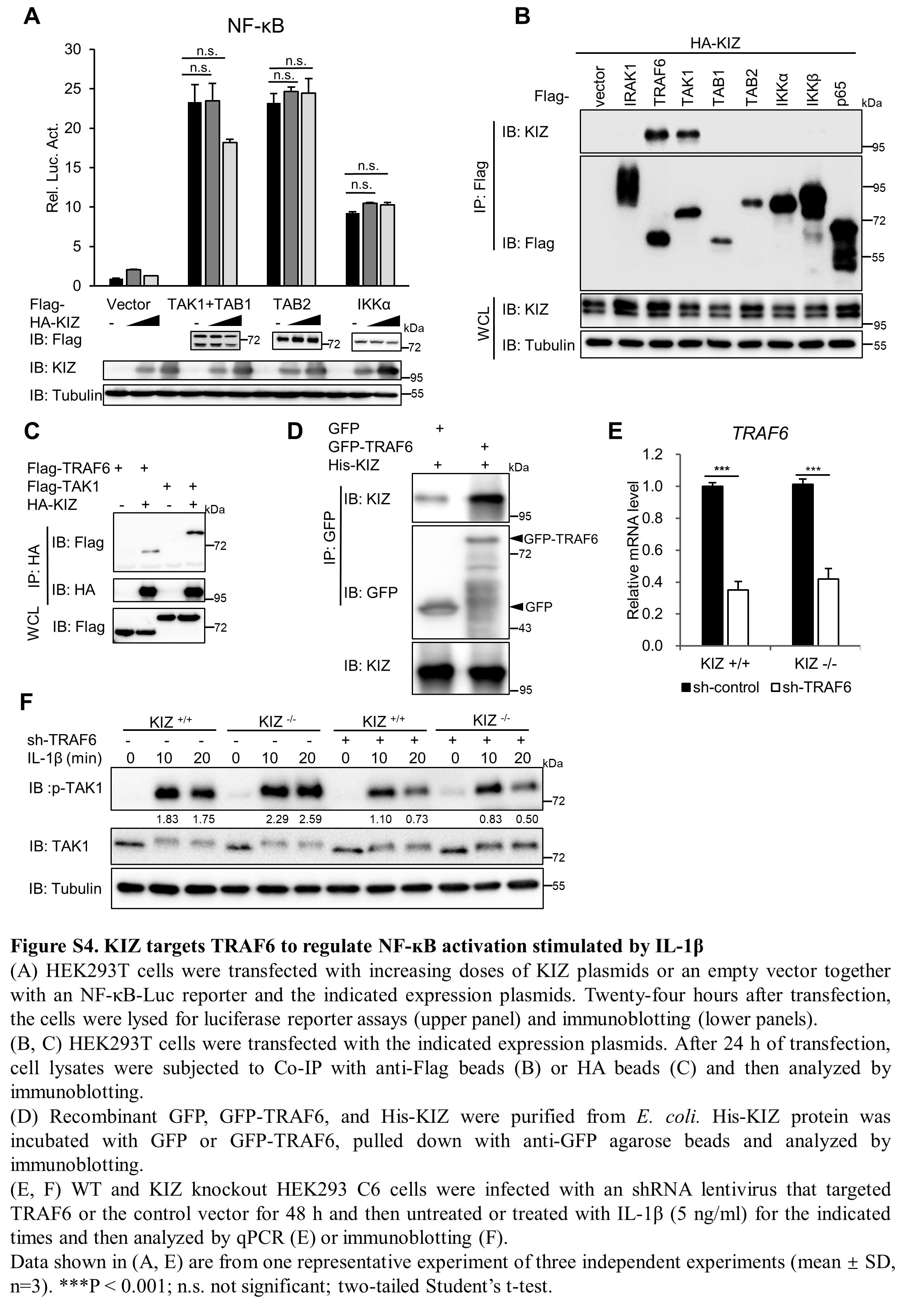

Supplement: Supplementary file 3 [file Image4.TIF]

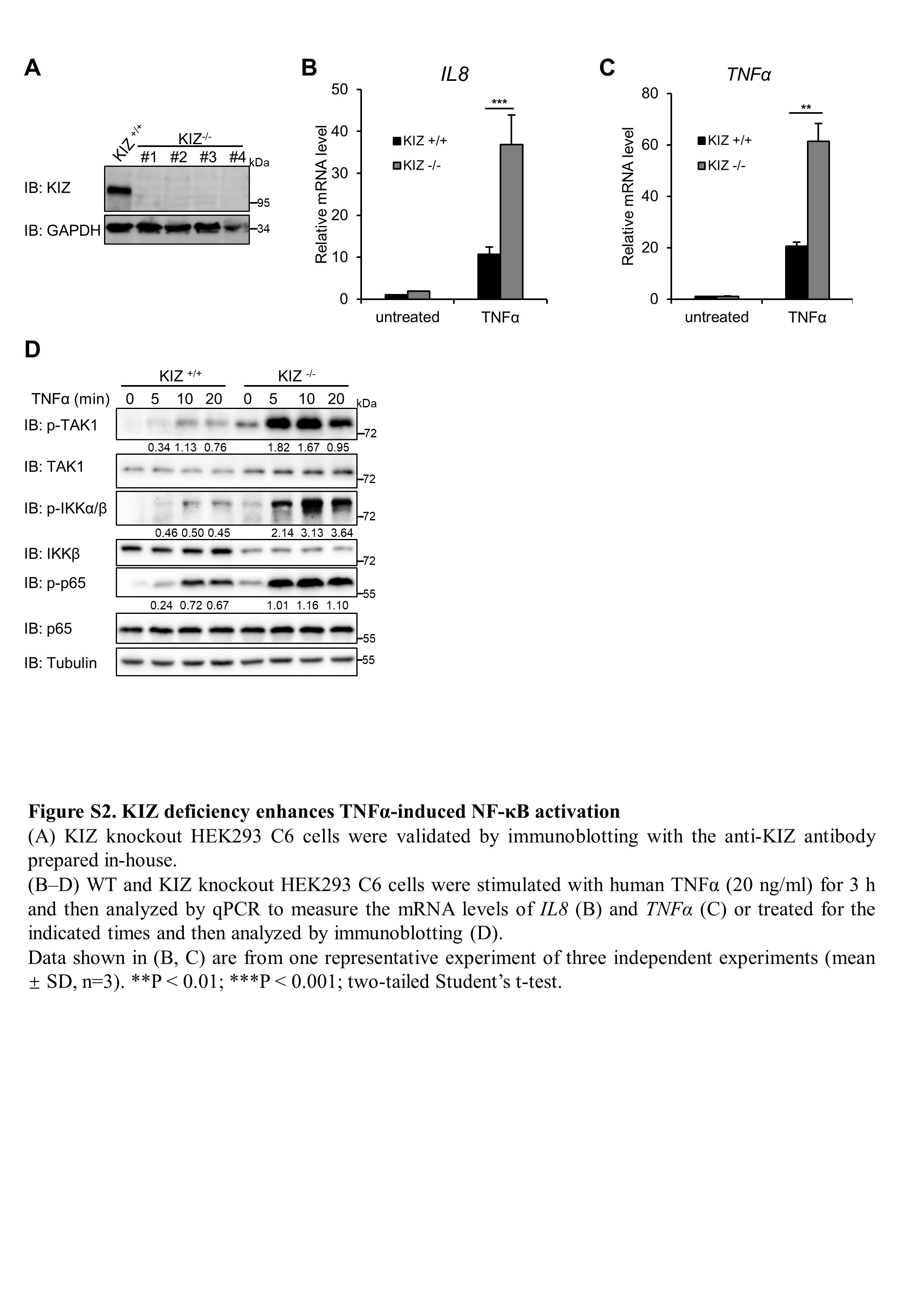

Supplement: Supplementary file 4 [file Image2.TIF]

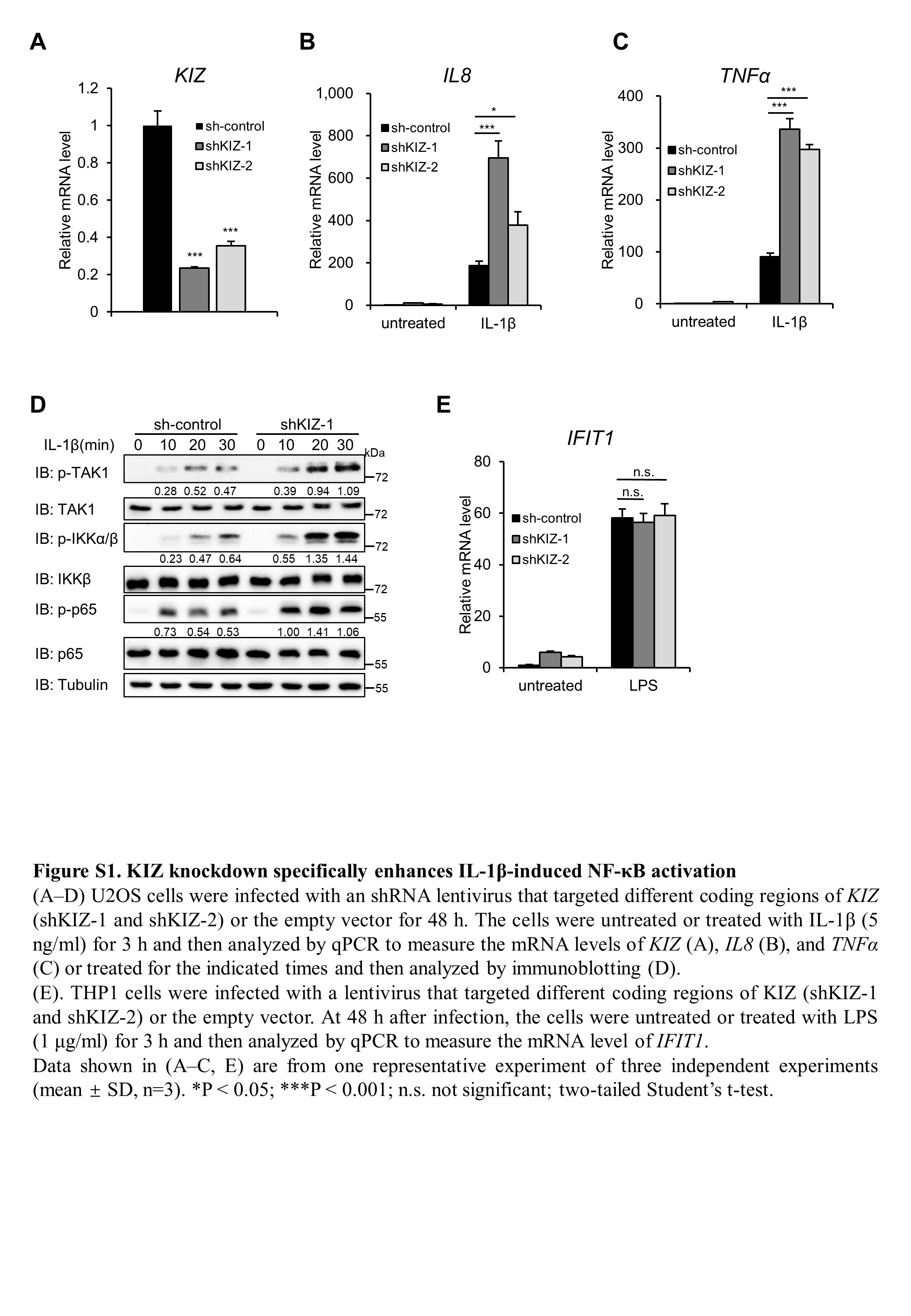

Supplement: Supplementary file 5 [file Image1.TIF]

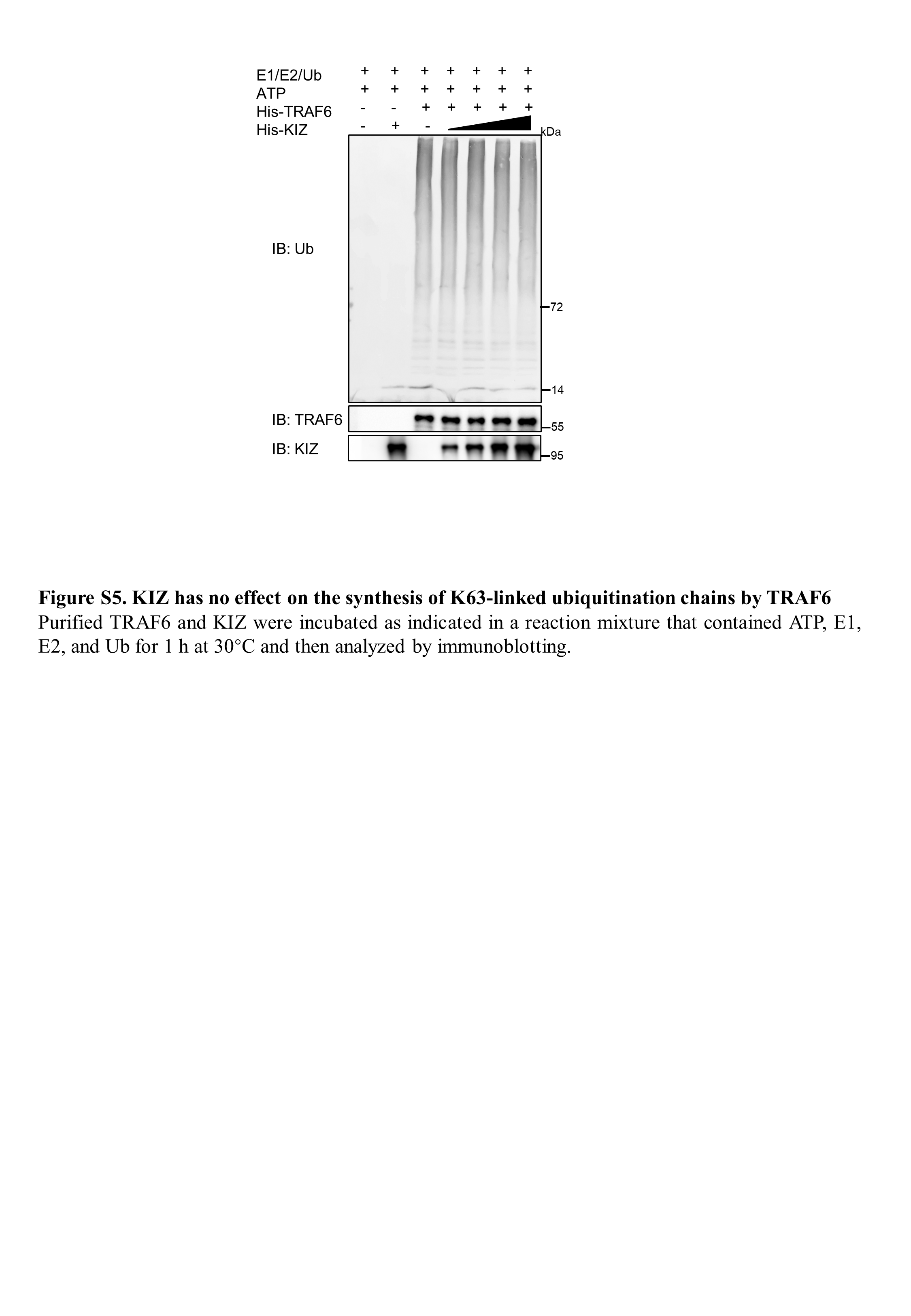

Supplement: Supplementary file 6 [file Image5.TIF]
